# A Comparative Analysis on Assessment of Land Carrying Capacity with Ecological Footprint Analysis and Index System Method

**DOI:** 10.1371/journal.pone.0130315

**Published:** 2015-06-29

**Authors:** Yao Qian, Lina Tang, Quanyi Qiu, Tong Xu, Jiangfu Liao

**Affiliations:** Key Lab of Urban Environment and Health, Institute of Urban Environment, Chinese Academy of Sciences, Xiamen, China; Kenya Medical Research Institute—Wellcome Trust Research Programme, KENYA

## Abstract

Land carrying capacity (LCC) explains whether the local land resources are effectively used to support economic activities and/or human population. LCC can be evaluated commonly with two approaches, namely ecological footprint analysis (EFA) and the index system method (ISM). EFA is helpful to investigate the effects of different land categories whereas ISM can be used to evaluate the contributions of social, environmental, and economic factors. Here we compared the two LCC-evaluation approaches with data collected from Xiamen City, a typical region where rapid economic growth and urbanization are found in China. The results show that LCC assessments with EFA and ISM not only complement each other but also are mutually supportive. Both assessments suggest that decreases in arable land and increasingly high energy consumption have major negative effects on LCC and threaten sustainable development for Xiamen City. It is important for the local policy makers, planners and designers to reduce ecological deficits by controlling fossil energy consumption, protecting arable land and forest land from converting into other land types, and slowing down the speed of urbanization, and to promote sustainability by controlling rural-to-urban immigration, increasing hazard-free treatment rate of household garbage, and raising energy consumption per unit industrial added value. Although EFA seems more appropriate for estimating LCC for a resource-output or self-sufficient region and ISM is more suitable for a resource-input region, both approaches should be employed when perform LCC assessment in any places around the world.

## Introduction

No matter how advanced science and technology becomes, human beings consistently rely on natural resources for survival and living. Expansive urbanization associated with rapid industrialization places enormous pressure on the Earth’s resources, and humans’ requirements for resources have surpassed the planet’s regeneration capacity since the 1970s [1]. Unfortunately, the high ecological pressure in urban areas and almost fully loaded land carrying capacity are even more troublesome as cities continue to experience population expansion, consumption growth, resource overuse, waste and emission accumulation, et al [2]. Thus, it is essential to determine land carrying capacity (LCC) to ensure the safety of ecosystems and their sustainable development, or at least to slow down the degradation of natural capital. Currently, more and more regional science programs have been devoted to study the relationship between human beings and land-use situations. The United States’ NASA Land-Cover and Land-Use Change Program was designed to improve the understanding of human interactions with the environment. It is focused on providing foundational knowledge of sustainability, vulnerability, and resilience of land use and on addressing issues related to land-cover and land-use changes for the purpose of human welfare [3].

Due to prodigious changes in land use, research activities on LCC have experienced a significant shift from land population carrying capacity to land integrated carrying capacity [4, 5]. In the late 1790s, a distinguished demographer, Thomas Robert Malthus, first described the relationship between population explosion and the Earth’s carrying through Malthus’ population theory, making a significant contribution to human demography, modern evolutionary biology, ecology [6, 7]. The concept of LCC originates from the book “Road to Survival” by William Vogt, who precisely defined LCC from the point of view of the human population in 1948 [8]. Over the past few decades, research has extended to comprehensively assess the impacts of human activities on LCC. After assessing what population size could be continually supported in a specific area, many studies proposed land function-oriented research for improving LCCs [9]. Here we refer LCC as the maximum human population a given region can support under a certain level of economic development and environmental conditions.

There are several ways to calculate LCC, but changing land-use patterns caused by advancing modern lifestyles have complicated the calculation procedure. Due to expansive urbanization, main industries, human population, and wealth are concentrated in city centers, and a majority of human populations occupies a small amount of land area, the locals tend to lose sight of the space and significance for non-commercial agricultural production and ecological protection [10]. Thus, the concept of agricultural sustainability was integrated into LCC. And it was suggested to improve LCC from feedback of measuring the condition of agricultural sustainability (e.g., the maximum level of sustainable exploitation of human resources) [11]. Noticing the correlation of land, population, and agriculture with environmental degradation, Komatsu et al. (2005) selected villages in Inner Mongolia to investigate the relationship of combating desertification and agricultural sustainability, aiming to evaluate the land conversion policy’s influence on the supply-demand balance in rural communities [12]. Agro-ecological zoning methodology originated in the 1970s and was applied as a system to evaluate land for rain-fed and irrigated agriculture, forestry, and grazing. This methodology has been developed by the United Nation’s food and agriculture organization (FAO) to assist with land resource assessments for better management and monitoring of these resources. In particular, based on the land productivity potential, such systems are commonly used in developing countries in order to assure food security [13].

Two other commonly used approaches developed in recent decades are ecological footprint analysis (EFA) and the index system method (ISM). Proposed by Rees in 1992 and improved by him and his Ph.D. student, Wackernagal, in 1994, EFA explains the relationship between local inhabitants and land resources. EFA is defined as a measure of human requirements of the biosphere determined by calculating the biologically productive land and water area, including renewable resource consumption, infrastructure construction, and emissions from carbon oxidation caused by the burning of fossil fuels (excluding ocean absorption) [14–16]. In addition, EFA contributes greatly to urban policies and communication, particularly in western cities [17]. Some researchers have applied the bottom-up EFA to urban consumption at a metropolitan scale and found that food, transportation, and buildings were the largest components of the footprint [18]. In order to select useful ecological indicators based on scientific validity and policy utility, Blomqvist proposed a set of principles, including the consideration of both ecological foundations and common sense when making sustainability goals [19]. After Ma determined the five major factors influencing the national ecological footprint (gross domestic product, urbanization, distribution of income, export dependence, and service intensity) [20], ecological footprint calculations were improved by the application of a support vector machine. In contrast, ISM is used as a tool to comprehensively assess LCC and contains multiple factors, including society, economy, production, environment, resources, etc. Wei et al. (2014) developed an index system to assess LCC by the “drive force-pressure-state-response-control” conceptual model. And they concluded that pressure from socio-economic development decreased the LCC value in several coastal cities [21].

The primary goal of this paper is to further compare the two LCC-evaluation approaches above, namely EFA and ISM. The study intended to answer the following questions: which method is suited for determining future sustainable land use? Which factors contribute to LCC and exert a major effect on it? Will ecosystems suffer from human activities in areas where natural land has been converted into other land types (e.g., agricultural land, aquaculture grounds, industrial zones, or urban areas)?

## Methods and Materials

### Study area

The study area is sub-provincial region, Xiamen City, in Fujian Province, located in the southeast coastal of China. Xiamen City consists of an island and mainland, with a total area of 1,699 km^2^ [22]. Over the last few decades, suburbanization has altered the land-use pattern in this region. Urbanized land has expanded from the island to the mainland, which has led to an increase in built-up land from 18.3% in 2000 up to 31.2% in 2012 and a decrease in agricultural land from 66.9% in 2000 down to 57.3%. Adjusting the balance of social development, population growth, and ecological protection proves to be an important concern in Xiamen City.

The aim of LCC assessment in this region is to predict the long-term influences of human activities on land resources and propose effective land management strategies. Such an aim has regional representative meaning because Xiamen City is the core of the Western Taiwan Straits Economic Zone as well as a Model livable City in China.

### Ecological footprint analysis

Ecological footprint analysis is known as an effective tool for measuring the sustainable use of natural resources and a land’s ability to support human beings [23]. On the basis of different ecosystem service functions and production characteristics, land is divided into six categories [24]. Population consumption and waste emissions by corresponding land areas can be normalized so that different land types can be compared with each other. In general, EFA calculations contain two variables, including ecological footprint and biocapacity, which represent the demand and supply, respectively. This method can be used to determine the balance of ecological deficits and ecological surpluses in a time series to estimate LCC, based on which further analysis is made to examine if a city is moving toward or away from sustainability [25]. Along with pioneering studies, researchers and organizations (e.g., the World Wild Fund for Nature) have revealed wide applications of EFA and periodically publish ecological footprint reports to determine ecological footprints’ impacts on different economic zones associated with various governmental and non-governmental agencies (e.g., the Organization for Economic Cooperation and Development, the Association of Southeast Asian Nations, BRICs, and some African countries) [26].

The calculation process involves four steps. First, one calculates the per capita annual consumption of the main resources with Eq (1):

$$C_{i}=\left(P_{i}+I_{i}-E_{i}\right)/p_{i}$$ (1)

where, *i* is the consumption item, *C*_*i*_ (kg) is annual per capita consumption, *P*_*i*_ (kg) is annual production, *I*_*i*_ (kg) is annual imports, *E*_*i*_ (kg) is annual exports, and *p*_*i*_ is population.

Second, the yield factors *y*_*j*_ and equivalence factors *e*_*j*_ are imported (Table 1). Based on net primary productivity (NPP) from MODIS data with 1km resolution in 2001 and areas of different land-use types, Liu calculated yield factors at provincial level in China [27]. In this paper, we use specific yield factors that reflect ecosystem productivities in Fujian Province. This factor enables the comparison among all the types of biologically productive land. Equivalence factors were introduced by Wackernagel in 1999 in order to making biologically productive lands more comparable among different regions [28]. This factor is the average ratio of the production of a certain biologically productive land and that of the same land on a global scale.

**Table 1:** The values of yield factors and equivalence factors of different land-use types [27–28].

Land-use types	Yield factors	Equivalence factors
**Arable land**	1.01	2.8
**Forest land**	1.57	1.1
**Pasture land**	3.17	0.5
**Fishery ground**	3.17	0.2
**Built-up land**	1.01	2.8
**Fossil energy land**	0	1.1

Next, the per capita ecological footprint (PEF) and per capita biocapacity (PBC) are calculated with Eqs 2–4. In addition, 12% of the total land use is set aside for biodiversity protection [29, 30].

$${aa}_{i}=C_{i}/W_{i}$$ (2)

$$\text{PEF}=\sum \left(e_{j}\times \sum {aa}_{i}\right)$$ (3)

$$\text{PBC}=\left(1-0.12\right)\times \sum \left(a_{j}\times y_{j}\times e_{j}\right)$$ (4)

where, *aa*_*i*_ (ha) is the per capita actual biologically productive land of item *i*, *W*_*i*_ (kg/ha) is the yield of item *i*, PEF (ha) is the per capita ecological footprint, PBC (ha) is the per capita biocapacity, *j* is the type of biologically productive land, and *a*_*i*_ is the total area of a type of biologically productive land.

Finally, the ecological surplus and ecological deficit are calculated. If the value of PBC is larger than PEF, this biologically productive land exhibits an ecological surplus. Otherwise, it exhibits an ecological deficit.

We collected data to evaluate LCC for Xiamen City between 2000 and 2012. EFA calculation requires detailed resource data, including biotic resources (12 items) and energy (9 items), and all the data can be found in the yearbooks of the Xiamen Special Economic Zone (2000–2012) and other references (Table 2).

**Table 2:** Land-use classification and consumption items.

Land-use types	Purposes	Consumption items	Product types
**Arable land**	Supplying grain crops and economical crops	Crops	Biotic resources: agricultural primary products
	Supplying grain crops and economical crops	Oil plants	Biotic resources: agricultural primary products
	Supplying grain crops and economical crops	Vegetables	Biotic resources: agricultural primary products
	Supplying grain crops and economical crops	Poultry	Biotic resources: livestock products
	Supplying grain crops and economical crops	Pork	Biotic resources: livestock products
	Supplying grain crops and economical crops	Eggs	Biotic resources: livestock products
**Forest land**	Supplying woods and forestry products	Fruits	Biotic resources: agricultural primary products
	Supplying woods and forestry products	Tea	Biotic resources: agricultural primary products
**Pasture land**	Supplying livestock farming and animal products	Beef	Biotic resources: livestock products
	Supplying livestock farming and animal products	Mutton	Biotic resources: livestock products
	Supplying livestock farming and animal products	Milk and dairy	Biotic resources: livestock products
**Fishery ground**	Supplying aquiculture and aquatic products	Aquatic products	Biotic resources: agricultural primary products
**Built-up land**	Supplying human living space and public infrastructure space	Heating power	Energy: final energy
	Supplying human living space and public infrastructure space	Electricity supply	Energy: final energy
**Fossil energy land**	Absorbing CO_2_ from fossil fuels combustion	Raw coal	Energy: primary energy
	Absorbing CO_2_ from fossil fuels combustion	Coal products	Energy: primary energy
	Absorbing CO_2_ from fossil fuels combustion	Natural gas	Energy: primary energy
	Absorbing CO_2_ from fossil fuels combustion	Petroleum	Energy: primary energy
	Absorbing CO_2_ from fossil fuels combustion	Diesel oil	Energy: primary energy
	Absorbing CO_2_ from fossil fuels combustion	Liquefied petroleum gas	Energy: primary energy
	Absorbing CO_2_ from fossil fuels combustion	Fuel oil	Energy: primary energy

### Index System Method

As ISM can reflect various features of an evaluated object simultaneously, we developed a framework of the fuzzy comprehensive evaluation model that covers all the basic information and main characteristics of land and human activities [31]. To simplify the description of complex parameters, indices of *C*_1_ to *C*_20_ are substituted for the factors (Table 3). Those parameters mentioned above are categorized into three main headings: land social-developmental carrying capacity (*B*_1_), land ecological-environmental carrying capacity (*B*_2_) and land economic-productive carrying capacity (*B*_3_).

**Table 3:** ISM framework for assessing LCC.

Criterion layer B	Index layer C	Index types	Justification
Land social-developmental carrying capacity, *B*_1_	Population density, *C*_1_	negative	Higher rate, more crowed.
	Employment rate, *C*_2_	positive	Higher rate, more stable of society.
	Engel coefficient, *C*_3_	negative	Residents’ living standard <40%, rich; >60%, poor (Food and Agriculture Organization).
	Residential land use rate, *C*_4_	positive	Higher rate, more space for living.
	Year-end road area, *C*_5_	positive	Higher rate, more extensive infrastructure construction.
	Per capita arable land, *C*_6_	positive	Higher rate, less pressure between population and crop yields.
	Urbanization rate, *C*_7_	positive	Higher rate, higher degree of urbanization.
Land ecological-environmental carrying capacity, *B*_2_	Green coverage, *C*_8_	positive	Higher rate, higher degree of urban greening.
	Comprehensive utilization of industrial solid wastes, *C*_9_	positive	Higher rate, fewer problems of environmental pollution and human security.
	Urban industrial wastewater discharge compliance rate, *C*_10_	positive	Higher rate, fewer problems of water pollution.
	Environmental investment index, *C*_11_	negative	Higher rate, fewer environmental problems to be solved.
	Centralized sewage treatment rate, *C*_12_	positive	Higher rate, higher degree of ability on sewage treatment.
	Hazard-free treatment rate of household garbage, *C*_13_	positive	Higher rate, more garbage being disposed.
Land economic-productive carrying capacity, *B*_3_	GDP, *C*_14_	positive	Higher rate, higher standard of economic development.
	Industrial output, *C*_15_	positive	Higher rate, higher degree of industrial enterprise development.
	Proportion of tertiary industry, *C*_16_	positive	Higher rate, more optimized of industrial structure and advanced of science and technology.
	Total retail sales of social consumer goods, *C*_17_	positive	Higher rate, higher purchasing power of commodities and larger scale of retail market.
	Intermediate consumption in of primary industry, *C*_18_	negative	Higher rate, less consumption of products and service during producing and operating.
	Energy consumption per unit industrial added value, *C*_19_	negative	Higher rate, fewer energies being consumed in industrial activities.
	Effective irrigation area of arable land ratio, *C*_20_	positive	Higher rate, higher degree of intensive water utilization in agricultural activities.

The calculation of ISM also involves four steps. The first step is to select indicators. Each index should reflect the corresponding characteristics of a subsystem. Frequency analysis is adopted to pick out and filter indicators by referencing to related literature, and satisfactory indicators are chosen on the basis of theoretical analysis. Moreover, to strengthen practicability, we take those indicators that frequently appear in appraisal modules of cities (e.g., indicators of National Model City for Environmental Protection) and apply the following selection principles: (1) inclusive relations, repeatability, and intersectionality are avoided; (2) representative indicators are first taken into account; i.e., indicators that can reflect the primary problems between sustainable land resource usage and social economic development; (3) selected indicators are adjusted according to local conditions (e.g., resources, environment, and regional differentiation) [32].

The second step is to identify the criterion of this system. A standard grading is applied to categorize the scores into five value groups between 0 and 1, which represent different ranks [33]. A higher value corresponds to stronger LCC, which means that each subsystem has better LCC conditions and the degree of coordination between subsystems is higher (Table 4). In terms of social development, if the value approaches 1, then the level of technology, popularization of education, rate of employment, quality of life, and coverage of public utilities will have a high standard. With regard to the ecological environment, if the value approaches 1, the ecosystem becomes stable to the point where it scarcely suffers damages from human activities, and cities have strong abilities to accommodate pollutants caused by mankind; in addition, people have a high pollution management efficiency and a low investment in environmental protection. As for economic productivity, when the value nears 1, human productivity and consumption have low carbon emissions. In addition, the layout and pattern of urban land use tends to be optimal at this point. Lower values correspond to weaker LCC.

**Table 4:** A grading standard of LCC.

Score interval	Classification
0.8–1	Strongest
0.6–0.8	Strong
0.4–0.6	Medium
0.2–0.4	Weak
0–0.2	Weakest

The third step is to normalize the indicators. We assume that there are *m* indicators, where, *C*_*i*_ (1≤*i*≤*m*), and *n* schemes, where *B*_*j*_ (1≤*j*≤*n*), which constitute a judgement matrix **X** = (*X*_*ij*_)_*n*×*m*_. All of the indicators can be divided into two categories, positive indicators and negative indicators. Higher property values correspond to better conditions for positive indicators, whereas lower property values correspond to better conditions for negative indicators. After a dimensionless transformation, all the indicators become positive, and the decision matrix becomes dimensionless, that is, **Y** = (*Y*_*ij*_)_*n*×*m*_. The attribute-based data are normalized by the method of maximum and minimum values, which converts all the variables into dimensionless numbers; the value of *Y*_*ij*_ is between 0 and 1, where 1 is the optimal value and 0 is the inferior value. Eqs 5 and 6 are as follows.

For positive indicators,

$$Y_{ij}=\frac{X_{ij}-\underset{1\leq i\leq m}{\text{min}}X_{ij}}{\underset{1\leq i\leq m}{\text{max}}X_{ij}-\underset{1\leq i\leq m}{\text{min}}X_{ij}}\left(1\leq i\leq m,1\leq j\leq n\right)$$ (5)

For negative indicators,

$$Y_{ij}=\frac{\underset{1\leq i\leq m}{\text{max}}X_{ij}-X_{ij}}{\underset{1\leq i\leq m}{\text{max}}X_{ij}-\underset{1\leq i\leq m}{\text{min}}X_{ij}}\left(1\leq i\leq m,1\leq j\leq n\right)$$ (6)

where, *Y*_*ij*_ is the normalized value, *X*_*ij*_ is the original value, max *X*_*ij*_ is the original maximum value, and min X_*ij*_ is the original minimum value.

The next step is to assign weights to the coefficients. In ISM, the weights greatly contribute to the calculation. Here we use the method of mean squared error (MSE), which can reflect the discrete degree of random variables and is calculated with Eqs 7–9. The last step is to evaluate the integrated LCC, as shown in Eq 10.

$$\bar{E_{j}}=\frac{1}{n}\sum_{i=1}^{n}Y_{ij}$$ (7)

$$\sigma \left(E_{j}\right)=\sqrt{{\sum_{i=1}^{\text{n}}\left(Y_{ij}-\bar{E_{j}}\right)}^{2}}$$ (8)

$$W_{j}=\frac{\sigma \left(E_{j}\right)}{\sum_{i=1}^{\text{n}}\sigma \left(E_{j}\right)}$$ (9)

$$F_{i}=\sum_{i=1}^{\text{n}}Y_{ij}\times W_{j}$$ (10)

where, $\bar{E_{j}}$ is the average value of a random variable, *σ*(*E*_*j*_) is MSE, *W*_*j*_ is the weight coefficient, and *F*_*i*_ is the final value of LCC in a target year.

The data used for ISM calculation consists of 20 indicators that are divided into 3 categories and can be found from the yearbooks of the Xiamen Special Economic Zone (Table 3).

## Results

### Ecological deficit situation reflected with EFA

**Fig 1:**
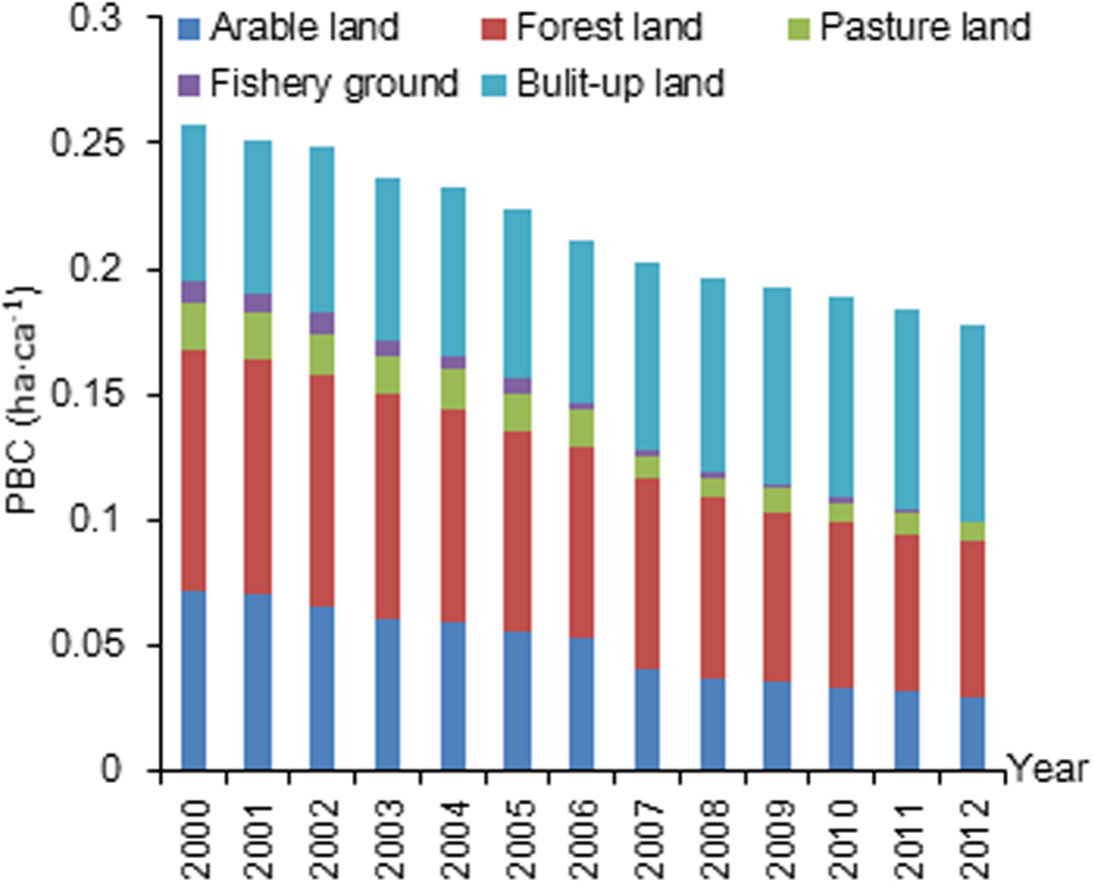
PBC of five biologically productive land types between 2000 and 2012 in Xiamen City.

**Fig 2:**
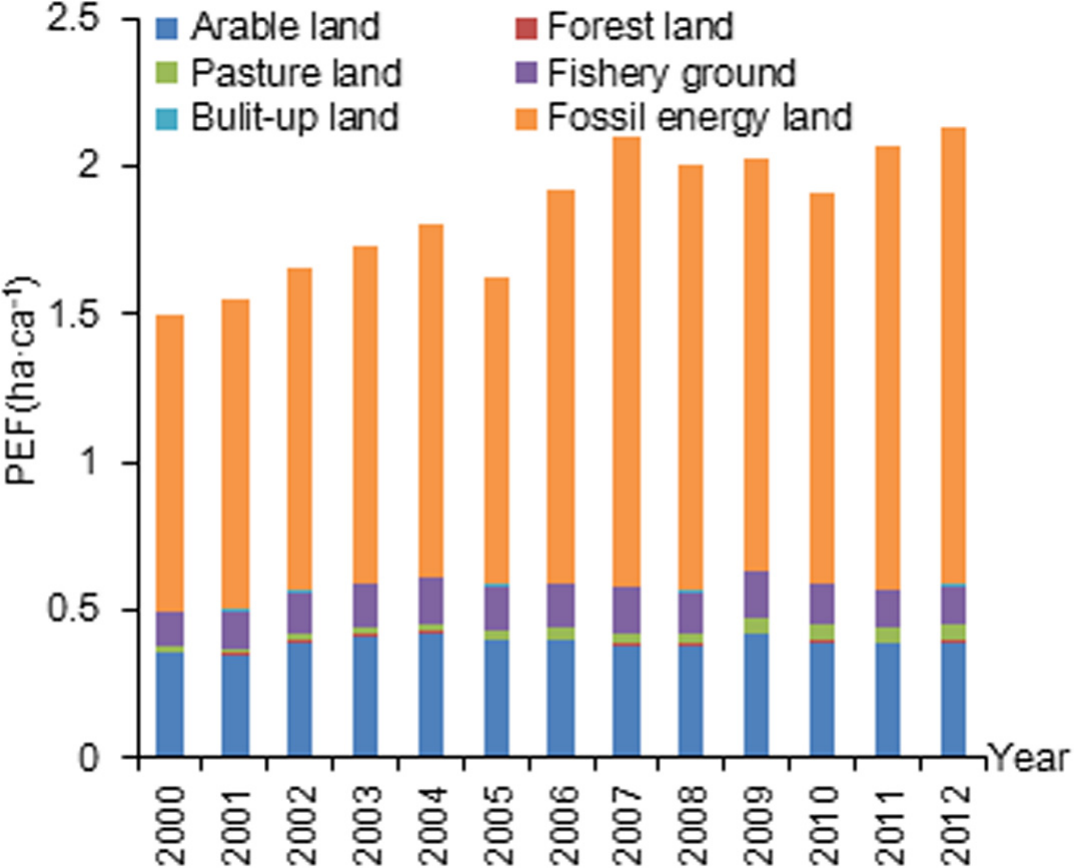
PEF of six biologically productive land types between 2000 and 2012 in Xiamen City.

**Fig 3:**
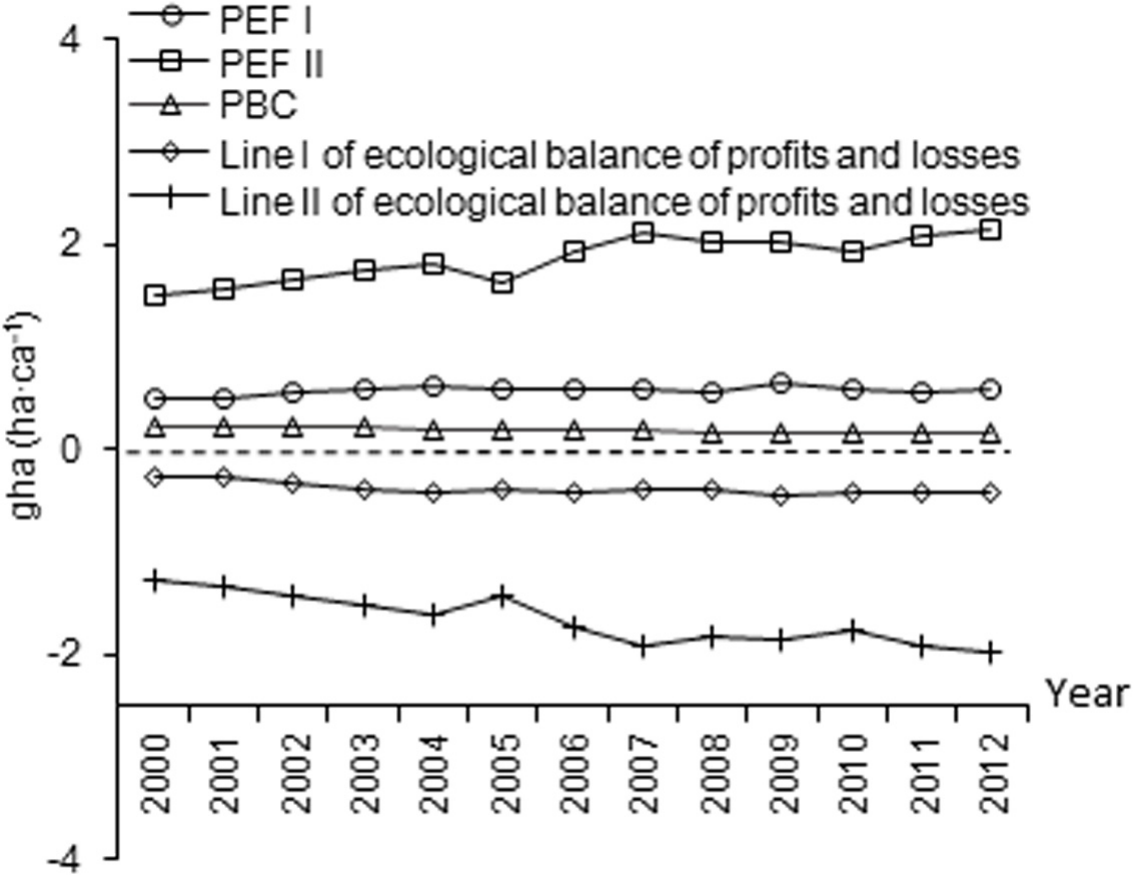
Ecological balance of profits and losses between 2000 and 2012 in Xiamen City. Energy footprint was not calculated in PEF I and line I of the ecological balance of profits and losses but in PEF II and line II of the ecological balance of profits and losses.

The total PBC decreased from 0.23 ha·ca^-1^ in 2000 to 0.16 ha·ca^-1^ in 2012 (Fig 1). Among the six land categories, arable land experienced the sharpest reduction in PBC (from 0.07 to 0.03 ha·ca^-1^over the 13 years). The total PEF increased from 1.49 to 2.12 ha·ca^-1^ between 2000 and 2012 (Fig 2). The PEF of fossil energy dominated the overall PEF and also experienced an increase (from 1.00 to 1.54 ha·ca^-1^ between 2000 and 2012). The line II of the ecological balance of profits and losses in Xiamen City declined from -1.26 to -1.96 ha·ca^-1^ over the 13-year period (Fig 3). When excluding fossil energy land, PEF I exhibits almost no change, and line I of the ecological balance of profits and losses remains stable.

**Fig 4:**
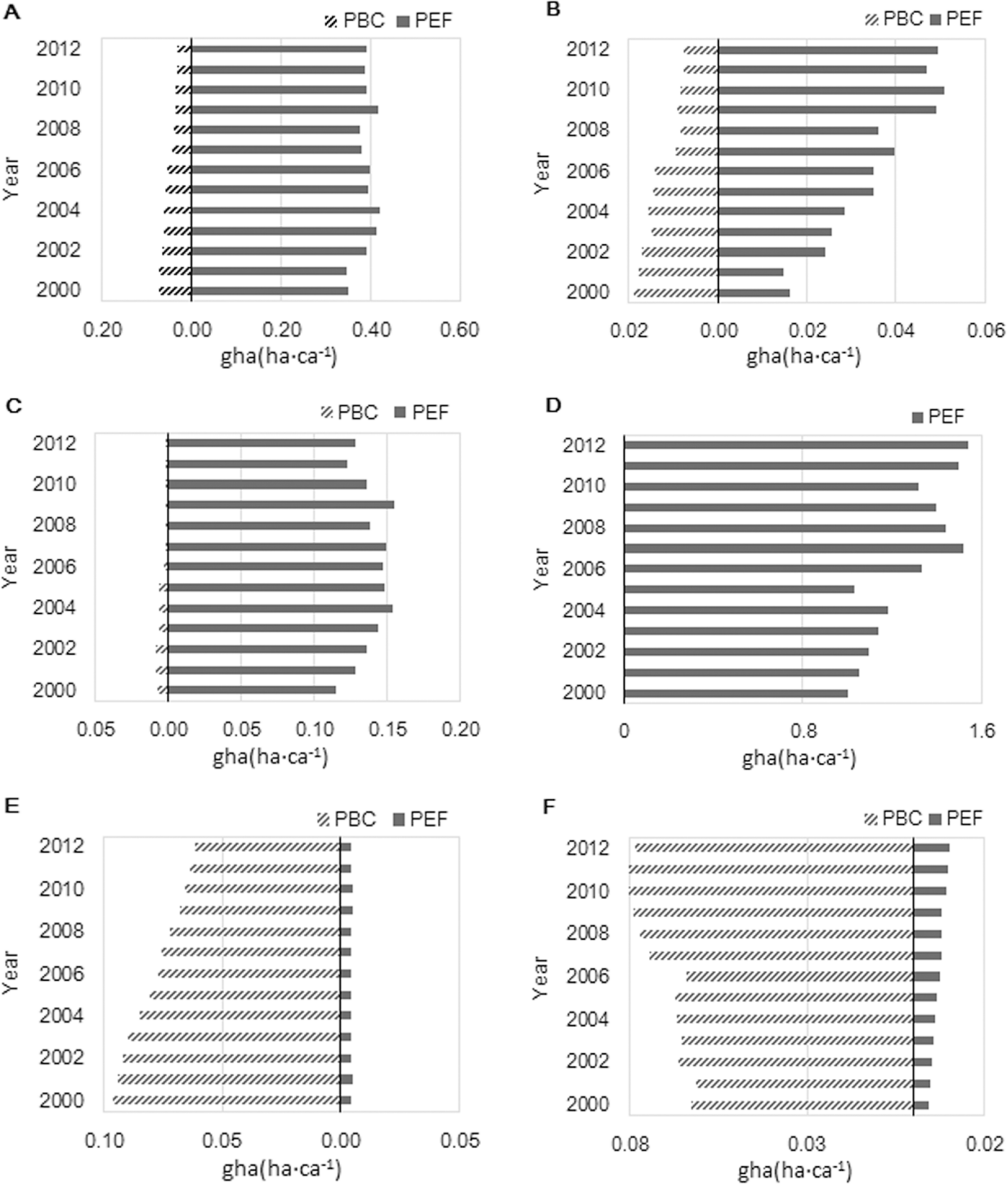
PBC and PEF of six biologically productive land types between 2000 and 2012 in Xiamen City. (A) Arable land; (B) Pasture land; (C) Fishery ground; (D) Fossil energy land; (E) Forest land; (F) Built-up land.

Four land-use types, including arable land, pasture land, fishery ground, and fossil energy land, had ecological deficit whereas the other two, including forest land and built-up land had ecological surplus (Fig 4). The temporal trends in ecological deficit/surplus were different among the six land-use types: the ecological deficits were getting greater over time for the four land-use types; the ecological surplus of forest land was getting smaller as its supply was declining; the surplus of built-up land was increasing because of faster increases in its supply (i.e., urbanization) than its demand.

**Fig 5:**
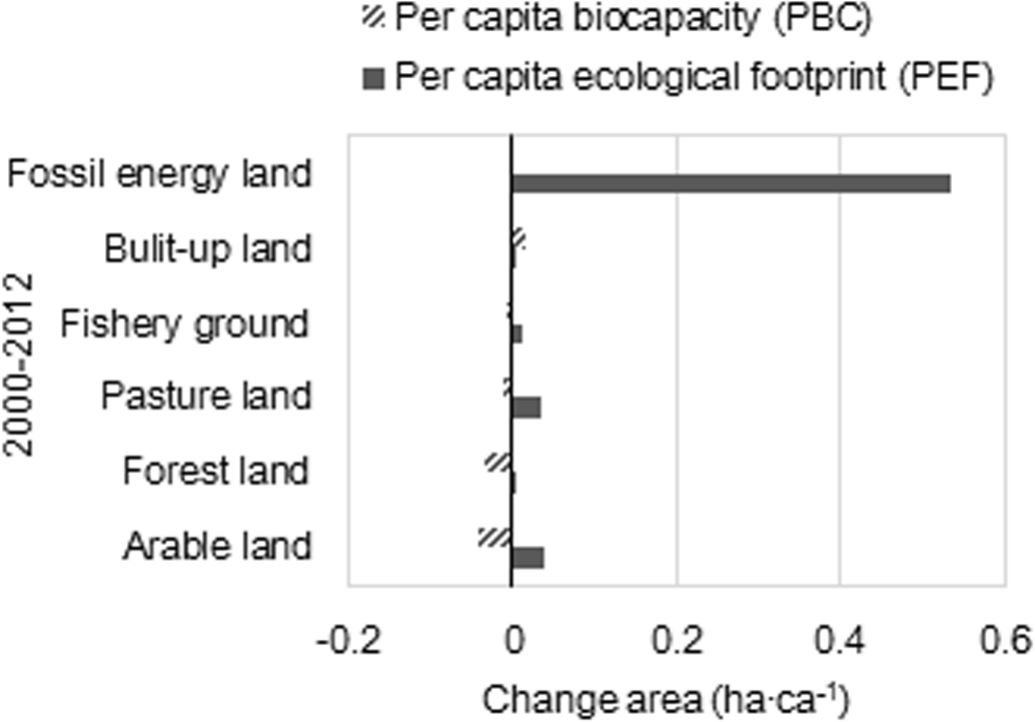
Change in area of six biologically productive land types between 2000 and 2012 in Xiamen City.

**Fig 6:**
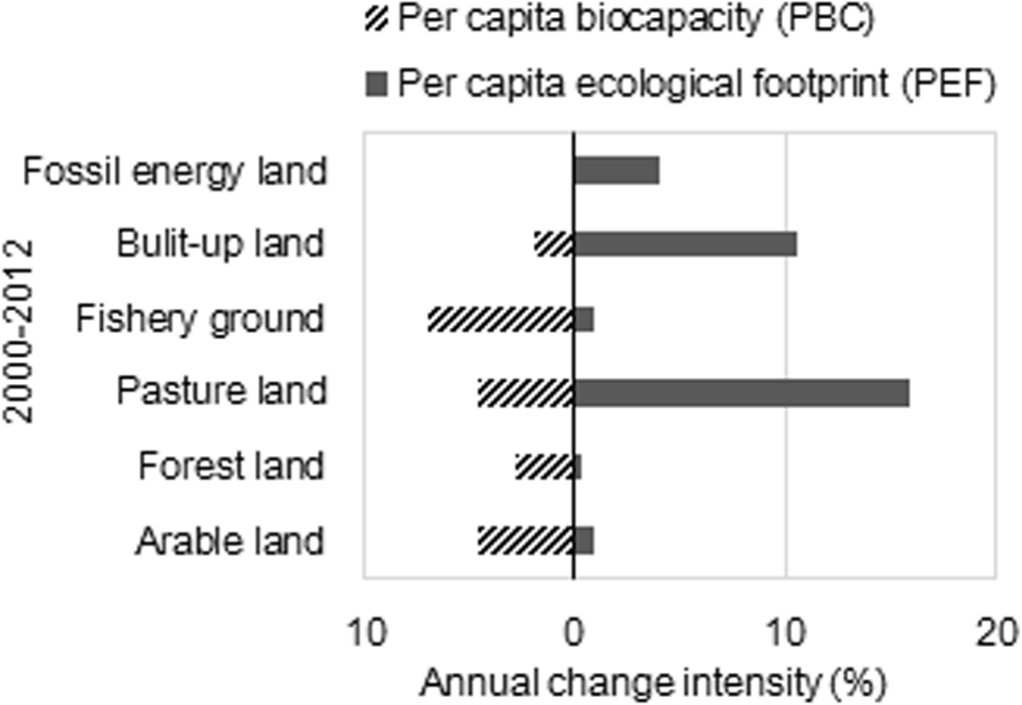
Annual change in intensity of six biologically productive land types between 2000 and 2012 in Xiamen City.

The dynamics trends in areas and intensity of the six biologically productive lands are diverse (Figs 5 and 6). Between 2000 and 2012, arable land lost 0.04 ha·ca^-1^ of PBC and had the same growth of PEF. Pasture land lost 0.01 ha·ca^-1^ of PBC, and had a growth of 0.03 ha·ca^-1^ in PEF, and exhibited an annual increasing rate of 15% in PEF, which represented the highest change among all land categories. The supply of fishery grounds decreased to 0.007 ha·ca^-1^ during these years, but the intensity of PBC decreased by 6.9% annually. Built-up land was the only one that exhibits area gains in PBC, meaning that the supply of built-up land increased. However, annual PEF intensity of built-up land increased by 10.6% but its annual PBC intensity decreased only by 1.9%. The PEF of fossil energy land increased to 0.54 ha·ca^-1^ in area and increased 4.1% in annual intensity. Fossil energy showed modest change intensity but a tremendous change in area gains, indicating that fossil energy land has been in high demand from 2000 to 2012 and exhibits an upward trend over time.

### Sustainability status revealed with ISM

The ISM-based LCC evaluation showed different results. The resulted weights of criterion layers ranked as follow: *B*_1_> *B*_3_> *B*_2_ (Table 5). As for the index weight, residential land use rate (*C*_4_) and per capita arable land (*C*_6_) contributed to a high proportion of *B*_1_. The hazard-free treatment rate of household garbage (*C*_13_) largely contributes to *B*_2_ and the environmental investment index (*C*_11_) contributes to it the least. The energy consumption per unit industrial added value (*C*_19_) contributes to *B*_3_, and the other factors are almost equal contributions.

**Table 5:** Index weights and normalized values (NV) of ISM.

Criterion layer (weight)	Index (Index weight)	NV(2000)	NV(2005)	NV (2012)	NV (2015)	NV (2020)	NV(2030)
***B***_**1**_ **(**0.339**)**	*C*_1_ (0.046)	1	0.861	0.621	0.519	0.345	0
	*C*_2_ (0.051)	0.090	0.099	0.553	0.470	0.647	1
	*C*_3_ (0.041)	0	0.315	0.432	0.550	0.700	1
	*C*_4_ (0.052)	0.025	0.641	0.398	0.373	0.542	0.880
	*C*_5_ (0.048)	0	0.081	0.401	0.473	0.649	1
	*C*_6_ (0.052)	1	0.532	0.283	0.270	0.101	0
	*C*_7_ (0.049)	0	0.273	0.675	0.707	0.767	1
***B***_**2**_ **(**0.328**)**	*C*_8_ (0.047)	0.082	0.147	0.450	0.495	0.663	1
	*C*_9_ (0.048)	0.265	0.433	0.820	0.851	0.911	1
	*C*_10_ (0.063)	0.868	0	0.917	0.936	0.957	1
	*C*_11_ (0.043)	0.611	0.376	0.535	0.548	0.592	1
	*C*_12_ (0.059)	0.075	0.450	0.805	0.847	0.932	1
	*C*_13_ (0.068)	0	0.237	0.884	0.942	0.977	1
***B***_**3**_ **(**0.333**)**	*C*_14_ (0.044)	0	0.030	0.139	0.212	0.374	1
	*C*_15_ (0.047)	0	0.129	0.436	0.483	0.655	1
	*C*_16_ (0.047)	0.114	0.036	0.311	0.571	0.643	1
	*C*_17_ (0.048)	0	0.077	0.416	0.470	0.646	1
	*C*_18_ (0.048)	0.950	0.681	0.451	0.486	0.324	0
	*C*_19_ (0.052)	0	0.259	0.704	0.759	0.852	1
	*C*_20_ (0.047)	0.492	1	0.719	0.418	0.337	0.037

Assuming every index follows a linear change over time, we made linear extrapolations to project its future values in 2015, 2020, and 2030, respectively (Table 5). Except for *C*_1_ and *C*_6_ (distributed in *B*_1_) and *C*_18_ and *C*_20_ (distributed in *B*_3_), the values of the remaining 16 indicators become higher with time and approach 1 in 2030.

**Fig 7:**
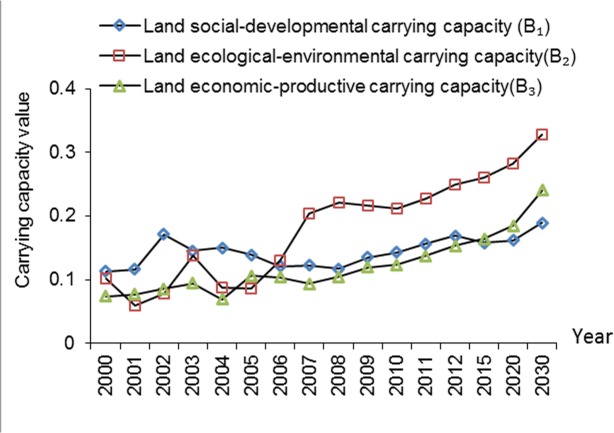
The social-development, ecological-environmental, and economic-productive land carrying capacity between 2000 and 2030 in Xiamen City.

The three subsystems all presented a rising trend in general, and the average increase rate ranked as *B*_2_ > *B*_3_ > *B*_1_ (Fig 7). In 2000, *B*_1_ was 0.11, *B*_2_ was 0.10, and *B*_3_ was 0.07. The value of *B*_2_ has increased distinctly since 2007 and it is predicted to grow continuously. *B*_1_ had the highest value in the first five years but would become the lowest in the future. In contrast, *B*_3_ had relatively steady increases over time.

**Fig 8:**
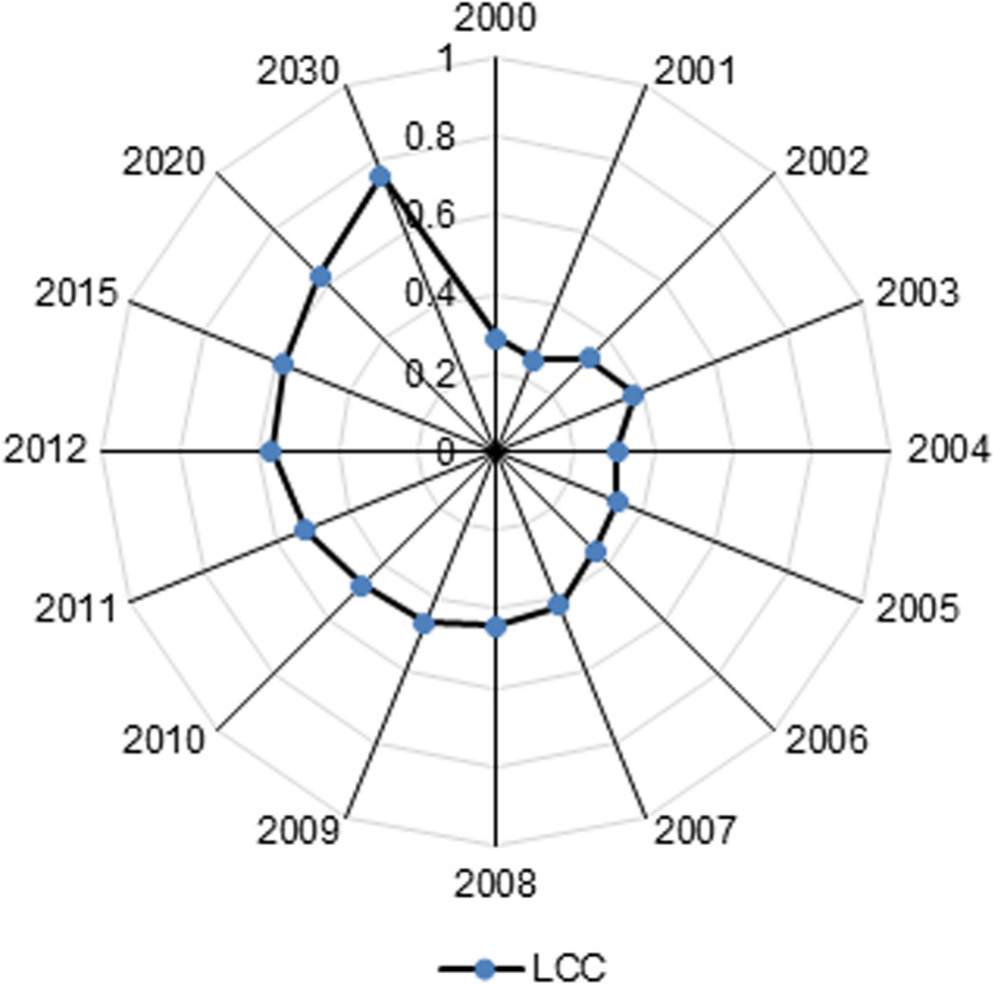
The integrated land carrying capacity between 2000 and 2030 in Xiamen City. LCC values calculated with ISM were used to assess the current situation and project the prospective state via linear extrapolation. The integrated value in each year also corresponds to the grading standard presented in Table 5.

These results show that LCC has experienced a continual increase between 2000 and 2012 (Fig 8). Furthermore, LCC is predicted to rise between 2015 and 2030 in Xiamen City. In the past, the average index value was 0.382, which indicating a “weak” carrying capacity, and the annual growth rate accounted for 7.3%. By 2012, the level of LCC has become “medium”, and the carrying conditions had slightly improved so that the average score was 0.58. Moreover, LCC is predicted to reach “strong”, with a value of 0.629 in 2020 and 0.758 in 2030.

## Discussion

### Limiting factors in sustainable development for Xiamen City

The EFA-based assessments explain that Xiamen City has experienced a constant ecological deficit between 2000 and 2012, representing a major challenge in its sustainable development. In this case, arable land made the highest contribution to the decline in overall PBC, and fossil energy land made the highest contribution to the rise in overall PEF for Xiamen City between 2000 and 2012. The energy footprint was the major cause of the ecological deficit. There are two major reasons that lead to the current high energy footprint. One is that there were limited energy sources but large energy consumptions in Xiamen City. Another is that the built-up area has increased tremendously between 2000 and 2012, which would trigger the increase in energy demand. Thus, it is important for the local policy makers to pay extra attention to control fossil energy consumption, protect arable land and forest land from converting into other land types, and reduce the speed of urbanization.

For ISM-based assessment, the integrated LCC increased continuously between 2000 and 2012, showing a good trend and well balance among social development, environmental treatment and economic growth. The in-depth analysis of the three factors of LCC reveals that ecological-environmental carrying capacity has experienced the greatest growth since 2006. This growth was the result of efforts Xiamen citizens and policy makers have made in ecological restoration and environmental protection, which was revealed by the indices of green coverage, the treatment of sewage, and the treatment of household garbage. On the contrary, the increasing population and decreasing arable land have contributed to the decline in social-development land carrying capacity between 2002 and 2008. In order to maintain social development, rural-to-urban immigration policy and arable land protection policy need to be strengthened.

### Suitability of EFA and ISM for LCC assessment

As LCC is a criterion that measures whether the region maintains sustainable development, it can be used to assist policy makers in urban land planning. The suitability of EFA and ISM for LCC assessment is worth discussing for various applications (Table 6).

**Table 6:** Suitability of EFA and ISM for LCC assessment.

Approaches	EFA	ISM
**Strengths**	Calculating 6 biologically productive lands.	Reflecting an overall trend as well as inner relationships of subsystem.
	Turning massive material datum and energy flows into a single, formal mode.	Data is generally available.
	Results are comparable among different levels.	Indicators are the relative numbers that strengthen comparability with each other.
**Limitations**	Any consumption of resources may be regarded as unsustainability.	Hard to choose indices reflecting practical significance.
	It may lose potential important variables.	Overly paying attention to other factors besides land.
	Data availability may be involved.	Avoiding interference caused by outliers.
**Applications**	Suitable area: Resource-output or self-sufficient regions with a relatively closed system.	Suitable area: Resource-input regions.
	Suitable issues: To examine the changes in different land categories and the trade-off tendency.	Suitable issues: To consider complex multi-factors and determine whether a region is under sustainable development overall.

EFA focuses on accessing whether an ecosystem is impacted negatively in the process of human production and consumption. EFA enables to estimate the carrying capacity of different biologically productive lands simultaneously, as well as characterize the dynamic processes of carrying capacity over time. The concept is quite simple and can be intuitively understood by ordinary people, managers and organizations [34]. In addition, the calculation of EFA is unified by integrating real data on massive material and energy flows into a single, formal mode. The results of EFA are comparable. EFA can show not only the level of LCC by comparing local states with the average national state, but also can reveal the level of LCC by comparing the national situation with the average worldwide situation. As a result, we may measure the conditions of national sustainable development and local lifestyles [35].

However, there are still weaknesses when using EFA for LCC assessment. EFA is a single aggregate static system [36]. Due to this limitation in calculation, any consumption of resources would be regarded as unsustainable from the viewpoint of the ecological footprint [25]. In addition, there are no consideration of other potential important variables for ecological services and resources consumption in the calculation, which may reduce the accuracy of the calculations [37]. The reliability of the results is closely related to the information availability. In our case study, there is a possibility of underestimating LCC with EFA. This underestimating resulting from that fossil energy land is a hypothetical land rather than a real one; thus, there is only a demand for it and no supply in the calculation. However, the calculation neglects the absorption of CO_2_ by other land types in accordance with the principle of space mutual exclusiveness, and this may lower the biocapacity. For instance, forest lands’ considerable capacity for CO_2_ assimilation is ignored based on the exclusive land ecological function adopted by the EFA, which only considers the supply of wood and other forestry products.

There are four merits of ISM for LCC assessment. Firstly, the results can reflect an overall trend as well as inner relationships of social development, environmental treatment, and economic growth subsystem. Secondly, all of the required data can be easily found in statistical yearbooks. However, EFA is more suitable to assess self-reliant regions within a closed system, such as islands [38]. Thirdly, relative values (e.g., percentage, growth rate, per capita, etc.) instead of real values are used to describe each index, which make the three subsystems are compatible [39].

The weakness of ISM is that the indices have weak practical significance, making the methodology lack persuasiveness. In addition, ISM pay too much attention to the factors such as living quality, environmental pollution and governance, economic growth, et al, which leads to the ignoring of direct land carrying capacity. Additionally, if outliers are present, it may draw false conclusions. For example, when values of one subsystem are extremely low but values of two other subsystems are large, the integrated value may be greater than the actual value. Another limitation of ISM is that LCC may be overestimated with ISM.

## Conclusions

EFA and ISM are both useful to evaluate LCC but each has special suitability depending on the region of application and the demands of policy makers. ISM is more suitable to resource-input regions, while EFA is appropriate for resource-output or self-sufficient regions. If policy makers wish to consider complex multi-factors, including social development, ecosystem health, and economic growth, in determining whether a region abides by sustainable development, ISM is more appropriate [40, 41]. However, if they wish to investigate the effects of different land categories on LCC, EFA is more suitable. In order to take full advantage of these two approaches, the combined use of them may be ideal to evaluate LCC.

The case study in Xiamen City demonstrates that LCC assessments with EFA and ISM are not only complement each other but also are mutually supportive. The decreasing supply of arable land has lowered PBC (assessed with EFA), and also reduced land social-developmental carrying capacity (assessed with ISM). These two assessments have proved that a decrease in arable land has negative effects on LCC for Xiamen City. In addition, high energy consumption has resulted in a low intensity of PEF (assessed with EFA) and a high value of energy consumption per unit industrial added value (assessed with ISM). Therefore, it is important to protect arable land and improve the efficiency of fossil energy for the purpose of promoting LCC in Xiamen City.

The situation of LCC in Xiamen City needs to be improved and major efforts have to be made jointly by policy makers, planners and designers [42]. From the viewpoint of reducing ecological deficits, it is important to control fossil energy consumption, protect arable land and forest land from converting into other land types, and reduce the speed of urbanization in Xiamen City; from the viewpoint of promoting sustainability, it is critical to maintain social development through controlling rural-to-urban immigration, protect the environment by increasing hazard-free treatment rate of household garbage, and promote economic growth while raising energy consumption per unit industrial added value.
